# Lincoln’s Electro-Therapeutics

**Published:** 1874-09

**Authors:** 


ELECTRO-THERAPEUTICS: A condensed manual of *Medical Electricity*. By D. F. Lincoln, M.D., Physician to the Department of Diseases of the Nervous System, Boston Dispensary. Philadelphia: Henry C. Lea. 1874. 12mo. Cloth; pp. 186. Price $1.50.

With the very general revival of the interest of the profession in the study and application of electro-therapia, comes the issue from the medical press of quite a number of monograms upon the subject. The one before us is very concise, suffiently complete and very practical, for the physician who is endeavoring to grope his way towards the electrical light.

